# Neural correlates of reward processing in adults with 22q11 deletion syndrome

**DOI:** 10.1186/s11689-016-9158-5

**Published:** 2016-07-15

**Authors:** Esther D. A. van Duin, Liesbet Goossens, Dennis Hernaus, Fabiana da Silva Alves, Nicole Schmitz, Koen Schruers, Therese van Amelsvoort

**Affiliations:** Department of Psychiatry and Psychology, Maastricht University, Maastricht, The Netherlands; Department of Psychiatry, Academic Medical Centre Amsterdam, Amsterdam, The Netherlands

**Keywords:** 22q11 deletion syndrome, Psychosis, Reward, COMT

## Abstract

**Background:**

22q11.2 deletion syndrome (22q11DS) is caused by a microdeletion on chromosome 22q11.2 and associated with an increased risk to develop psychosis. The gene coding for catechol-O-methyl-transferase (COMT) is located at the deleted region, resulting in disrupted dopaminergic neurotransmission in 22q11DS, which may contribute to the increased vulnerability for psychosis. A dysfunctional motivational reward system is considered one of the salient features in psychosis and thought to be related to abnormal dopaminergic neurotransmission. The functional anatomy of the brain reward circuitry has not yet been investigated in 22q11DS.

**Methods:**

This study aims to investigate neural activity during anticipation of reward and loss in adult patients with 22q11DS. We measured blood-oxygen-level dependent (BOLD) activity in 16 patients with 22q11DS and 12 healthy controls during a monetary incentive delay task using a 3T Philips Intera MRI system. Data were analysed using SPM8.

**Results:**

During anticipation of *reward*, the 22q11DS group alone displayed significant activation in bilateral middle frontal and temporal brain regions. Compared to healthy controls, significantly less activation in bilateral cingulate gyrus extending to premotor, primary motor and somatosensory areas was found.

During anticipation of *loss*, the 22q11DS group displayed activity in the left middle frontal gyrus and anterior cingulate cortex, and relative to controls, they showed reduced brain activation in bilateral (pre)cuneus and left posterior cingulate.

Within the 22q11DS group, COMT Val hemizygotes displayed more activation compared to Met hemizygotes in right posterior cingulate and bilateral parietal regions during anticipation of reward. During anticipation of loss, COMT Met hemizygotes compared to Val hemizygotes showed more activation in bilateral insula, striatum and left anterior cingulate.

**Conclusions:**

This is the first study to investigate reward processing in 22q11DS. Our preliminary results suggest that people with 22q11DS engage a fronto-temporal neural network. Compared to healthy controls, people with 22q11DS primarily displayed reduced activity in medial frontal regions during reward anticipation. COMT hemizygosity affects responsivity of the reward system in this condition. Alterations in reward processing partly underlain by the dopamine system may play a role in susceptibility for psychosis in 22q11DS.

## Background

Psychotic disorders, including schizophrenia, are potentially devastating lifelong illnesses that are disabling and costly to patients, families, communities and healthcare systems. Symptoms typically emerge during late adolescence, and the estimated lifetime prevalence and incidence is approximately 0.3–0.7 % [1].

Treatment advances in these heterogeneous disorders have been limited by insufficient mechanistic understanding of the underlying pathophysiology. Thus far, pharmacological treatments have been based on the premise of disrupted dopaminergic neurotransmission, but the exact nature of dopamine (DA) dysregulation remains complex [2].

One of the more recent theories of psychosis suggests that an aberrant brain reward system could explain some of the disorder’s clinical symptoms [3]. Anticipation of reward represents motivational behaviour or drive (“wanting”), which is associated with activation of the typical cortical-basal ganglia circuit [4] and particularly modulated by dopamine in the ventral striatum. Consequently, dopamine depletion results in lack of motivational drive, apathy [5] and reduced brain activity in striatum and cingulate gyrus during anticipation of reward [6], whereas amphetamine-induced dopamine release in striatal brain regions has been associated with pleasant emotions of anticipation [5, 7]. Over recent years, several functional magnetic resonance imaging (fMRI) studies have demonstrated alterations in the brain reward network in patients with, and at clinical high risk for, psychosis, primarily in the striatal motivational system [8–13].

One of the most important proteins that regulate extracellular brain dopamine concentrations is catechol-O-methyl-transferase (COMT), an enzyme catabolising released dopamine in cortical, particularly prefrontal, areas [14]. A functional single nucleotide polymorphism, Val158Met of the COMT gene (Val/Met), has been suggested to lead to a 40 % reduction in enzyme activity and has been shown to affect cortical DA metabolism levels, with Val carriers displaying lower extracellular DA levels than Met-carriers [15]. This polymorphism contributes to measurable individual differences in human cognitive function [16–18]. Moreover, fMRI studies in healthy participants have shown that frontal and striatal activation during anticipation of reward is dependent on COMT genotype with Met homozygotes showing larger brain response than Val homozygotes [19, 20].

Interestingly, the gene for COMT is located at chromosome 22q11.2, a chromosomal region that has received an interest from psychiatric geneticists for over 20 years. A deletion at 22q11.2 is the first and only copy number variant unequivocally implicated in psychotic disorders: people with 22q11 deletion syndrome (22q11DS) carry a 25- to 30-fold increased risk of psychosis [21–23]. This shared genetic variant that greatly increases risk for psychosis makes individuals with 22q11DS a relatively homogeneous population to study psychotic vulnerability. Thus, 22q11DS can provide unique insights into risk and protective factors for psychotic vulnerability that not only benefit patients with 22q11DS but also could help patients with psychosis that do not have this particular deletion.

COMT haplo-insufficiency in 22q11DS has been suggested as one explanation for the increased susceptibility for psychosis in 22q11DS. Indeed, it has been demonstrated that people with 22q11DS have reduced COMT gene expression [24, 25], enzyme activity [25] and alterations in dopaminergic neurotransmission [26, 27]. Because of a paucity of dopamine transporter expression in the frontal lobe, dopamine metabolism is largely dependent on COMT in frontal brain regions. Therefore, effects of reduced COMT gene dosage are expected to be most pronounced in frontal brain regions in subjects with 22q11DS [25, 28]. In addition, the Val/Met polymorphism may have a larger effect in 22q11DS because only one copy of the allele is present, and COMT Met hemi-zygotes may have extremely low COMT activity [15, 17, 25, 29, 30]. While COMT haplo-insufficiency has been proposed as one explanation for the increased risk of psychosis in 22q11DS, it should be noted that, overall, the association between COMT genotype and psychosis remains inconclusive [31–33].

The consequences of COMT haplo-insufficiency in humans with 22q11DS at a neuronal level, and how this relates to psychotic symptomatology is still unclear. More specifically, the effect of the 22q11.2 deletion and COMT haplo-insufficiency on reward processing is still unknown. We therefore explored for the first time reward processing in adults with 22q11DS using a reward anticipation fMRI paradigm. We hypothesized that adults with 22q11DS, because of their increased susceptibility for psychosis, would not recruit brain regions that would normally be recruited during motivational behaviour. In addition, we hypothesized that, in 22q11DS, brain activation during reward processing would be modulated by COMT Val/Met genotype.

## Methods

### Subjects

Adult individuals with 22q11DS (*n* = 16) were recruited through the Dutch 22q11DS family association and several Dutch Clinical Genetics Centres. Healthy volunteers (*n* = 12) were recruited by local advertisement as described previously and are partially overlapping with the healthy volunteers of our previous studies [6, 34]. The study was conducted at the Department of Psychiatry, Academic Medical Centre Amsterdam, The Netherlands, and was approved by the local Medical Ethics Committee. All participants were capable of giving written informed consent and did so, after receiving full information on the study. All individuals with 22q11DS were interviewed by a physician using a semi-structured psychiatric interview. Patients with 22q11DS with psychosis were all on anti-psychotic medication and two 22q11DS patients without psychosis were using selective serotonin reuptake inhibitors (SSRIs) at the time of testing (Table 1). None of the healthy participants had a history of psychiatric disorders, medical conditions affecting brain function and substance or alcohol abuse, and they were not using any medication at the time of testing.

**Table 1:** Characteristics of participants

	22q11DS (*n* = 16)	Controls (*n* = 12)	*p*
Age (SD)	28.2 (6)	29 (9.6)	0.79
Gender (M/F)	8/8	8/4	0.46
IQ (SD)	77 (10)	110 (10)	<0.001
Psychosis (Y/N)	5/11		
COMT genotype (Met/Val)	6/10		
PANSS total	45.5		
PANSS positive	8.4		
PANSS negative	13.9		
Medication (*n*)	Quetiapine (3)		
	Risperidone (1)		
	Lithiumcarbonate (1)		
	Paroxetine (1)		
	Methylphenidate (1)		
	Venlafaxine (1)		
	Clozapine (1)		
	Lamotrigine (1)		

The Positive and Negative Symptom Scale (PANSS) [35] was used to assess positive, negative and general psychopathology in the 22q11DS group. In addition, for assessment of intelligence quotient (IQ), we used the shortened Dutch version of the Wechsler Adult Intelligence Scale (WAIS-III-NL) consisting of five subtests: vocabulary, comprehension, similarities (verbal IQ), block design and object assembly (performance IQ) [36, 37]. For demographics and clinical variables, see Table 1.

### Genotyping

Blood samples were collected from all subjects with 22q11DS participants. DNA was isolated from blood using standard procedures (Gentra Technology, Qiagen). Genotyping using 5′-nuclease Taqman assays for allelic discrimination (Life Technologies, Foster City, California, USA) was carried out with a LC-480 384-well Lightcycler (Roche Diagnostics, Mannheim, Germany) [38]. COMT Val^158^Met (rs4680) genotype was determined with Taqman assay C.25746809 A/G (Life Technologies). The Lightcycler LC-480 Software release 1.5.0 was used to analyse end point fluorescence.

### FMRI task: monetary incentive delay

**Fig. 1:**
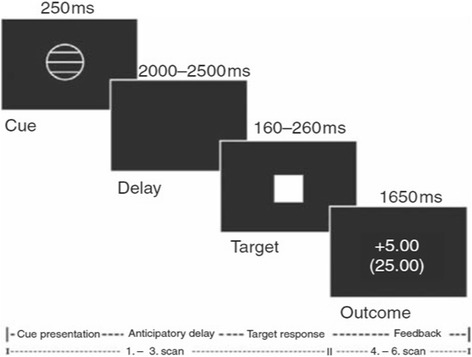
Monetary incentive delay task, structure for a representative trial

We used event-related fMRI to assess blood-oxygen-level dependent (BOLD) brain activation during the monetary incentive delay (MID) task (Fig. 1) [39]. In short, the MID task was used to evoke anticipation of potential monetary reward, loss or no consequential outcome. It consists of two sessions of 72 trials of 6 s, yielding a total of 144 trials and total duration of 14 min. During each trial, subjects were shown one of seven cues. Cues signaling reward were denoted by circles (*n* = 54), loss by squares (*n* = 54) and no monetary outcome by triangles (*n* = 36). The amount of money that subjects were able to win was indicated by one horizontal line (0.20 Euro), two lines (1.00 Euro) and three lines (5.00 Euros). Similarly, loss cues signalled the possibility of losing the same amounts of money. Subjects had to respond to the white target square that appeared for a variable length of time. To succeed in a trial, volunteers had to press the button during the time that the white square target was visible (target, 160–260 ms). Unlike the MID described by Knutson et al. [35], we did not pay the amount of money earned during the task; reward and loss was based on point scoring [6, 34].

### MRI data acquisition

FMRI data were collected using a 3T MRI Philips system equipped with a sense head coil as previously explained [6, 34]. The task stimuli were generated using e-prime software (SCOPE V2.5.4/Pentium). For the MID task 360 event related, transversal multi-slice T2*-weighted gradient-echo planar images (EPI) were acquired with echo time (TE) 30 ms, repetition time (TR) 2000 ms, 96 × 96 matrix, 35 slices, 3 × 3 mm in-plane resolution, slice thickness 3 mm with a 1-mm interslice gap, covering the entire brain. For anatomical localization, transversal high-resolution structural T1-weighted volumetric images were acquired in the same session, with full head coverage, using 150 contiguous slices (1-mm thick, with 0.89 × 0.89 mm in-plane resolution), a 256 × 256 × 124 matrix and a TR/TE of 24/5 ms (flip angle 45°, FOV 24 cm).

### FMRI data analyses

All functional and structural brain images were pre-processed with the researcher blind for group status, as previously explained [6].

#### FMRI data pre-processing

Slice time correction was used to adjust for time differences due to multi-slice image acquisition. The functional images were realigned to the first volume of the time series to correct for head movements. After co-registering functional images to the anatomical image, they were spatially normalized to the standard space of the Montreal Neurological Institute brain (MNI-brain). All functional images were sub-sampled to a voxel size of 2 × 2 × 2 mm. Normalized images were smoothed with a Gaussian kernel of 8-mm full width at half maximum.

#### FMRI data statistical analysis

The analyses focused on changes in blood-oxygen-level dependent (BOLD) contrast that occurred during anticipatory delay periods and were conducted using SPM8 (Wellcome Department of Cognitive Neurology, London, UK). The pre-processed fMRI data were analysed in the context of the general linear model (GLM) approach [40] using a two-level procedure.

At the first level, seven conditions (Reward_High_, Reward_Medium_, Reward_Low_, Neutral, Loss_High_, Loss_Medium_, Los_low_) were modelled by a boxcar function convolved with a hemodynamic response function. The movement parameters were included as confounds in the design matrix. Changes in the BOLD response were assessed using the estimated GLM parameters for the anticipation of potential monetary gain vs. anticipation of no monetary outcome (reward vs. neutral) and the anticipation of potential monetary loss vs. anticipation of no monetary outcome (loss vs. neutral). In the second level analysis, individual contrast images of the first level analysis were included in a two-sample *t* test to detect relevant brain activation in patients with 22q11DS and in healthy controls. Subsequently, within the 22q11DS group, effects of COMT genotype and PANSS scores on brain activation were tested. For the whole brain analysis, comparisons were corrected for multiple comparisons using family wise error correction (FWE_cor_) *p* < 0.05 at the cluster level (extent threshold of 10 voxels).

## Results

### Demographic characteristics

Patients with 22q11DS did not differ in age compared to healthy controls (22q11DS 28.2 years ± 6.0 vs controls 29 years ± 9.6, *p* = 0.79). Also, gender distribution was not significantly different between the two groups (22q11DS M/F ratio 8/8; controls M/F ratio 8/4; *p* = 0.46, Fisher’s exact test). 22q11DS patients and healthy controls differed in total IQ scores (HC 110 (10) and 22qDS 77 (10), *p* < 0.001). Within the 22q11DS group, 5 had a psychotic disorder, 6 were Val hemizygote and 10 were Met hemizygote (Table 1).

### Task performance

Repeated measures ANOVA showed no significant main effect of group (*p* = 0.25) on reaction time performance. Reaction times in healthy controls in reward (243.4 ms ± 29.3) and loss (250.7 ms ± 38.4) conditions did not differ from those in patients with 22q11DS (reward 231.8 ms ± 29.2; loss 230.1 ms ± 29.3). There was no main effect of incentive value (*p* = 0.26) on reaction time performance and no significant interaction effect (incentive value*group, *p* = 0.09).

### FMRI results

#### Patients with 22q11DS

During *anticipation of reward*, patients with 22q11DS significantly activated a large cluster (23,094 voxels) encompassing the bilateral middle frontal lobe and bilateral middle and superior temporal lobe (*p*_FWE_ < 0.001 corrected for multiple comparisons at cluster level, Table 2). During *anticipation of loss,* patients with 22q11DS showed activation in a cluster (19786 voxels) including the left middle frontal gyrus and the anterior cingulate cortex (*p*_FWE_ < 0.001, Table 2).

**Table 2:** Peak level coordinates in the significant^*^ cluster during anticipation of reward

Group	Brain structure		BA	MNI coordinates			*T* score
				x	y	z	
22q11DS	L	Hypothalamus	NA	−10	−6	−8	4.83
	R	Inferior frontal gyrus	47	26	18	−12	5.08
	L	Medial frontal gyrus	6	−10	−30	74	5.35
	L	Middle frontal gyrus	8	−24	20	48	4.53
	R	Middle frontal gyrus	10	34	50	0	4.53
	L	Middle temporal gyrus	21	−52	−46	4	4.37
	R	Middle temporal gyrus	21	54	−24	−12	4.64
	R	Putamen	NA	28	−10	12	4.64
	L	Superior temporal gyrus	39	−34	−58	28	4.52
	R	Superior temporal gyrus	41	56	−20	4	4.63
Controls	L	Cingulate gyrus	24	−4	−10	40	7.79
	R	Cingulate gyrus	24	4	−12	40	7.26
	R	Cingulate gyrus	23	4	−16	34	6.96
	R	Cingulate gyrus	23	4	−32	28	5.52
	R	Cingulate gyrus	24	2	−18	44	9.02
	R	Cingulate gyrus	23	4	−12	30	5.64
	R	Middle occipital gyrus	18	32	−88	−8	7.39
	L	Posterior cingulate	23	−2	−30	24	9.25
	R	Precentral gyrus	4	20	−28	72	6.22
	L	Precuneus	31	−8	−62	22	5.73
	R	Precuneus	31	20	−78	26	6.58
	R	Superior frontal gyrus	6	6	16	68	5.69
	L	Transverse temporal gyrus	41	−42	−30	12	6.08
22q11DS > controls	No significant results						
Controls > 22q11DS	L	Cingulate gyrus	24	−4	−12	38	3.10
	L	Cingulate gyrus	24	−8	−20	40	3.24
	R	Cingulate gyrus	24	4	−12	40	4.63
	R	Cingulate gyrus	23	4	−30	28	3.28
	R	Cingulate gyrus	24	2	−20	40	5.03
	R	Cingulate gyrus	31	12	−32	42	3.24
	R	Medial frontal gyrus	6	10	−12	74	3.66
	L	Paracentral lobule	5	−8	−44	50	3.12
	R	Paracentral lobule	4	6	−42	72	3.10
	R	Postcentral gyrus	4	12	−38	60	4.60
	L	Precuneus	31	−2	−70	24	3.31

* *p* < 0.001 FWE- corrected at cluster level

*L* left, *R* right, *BA* Brodmann area

Within the 22q11DS group without psychosis (*n* = 11), the same regions were found as in the total 22q11DS group during anticipation of reward (same peak clusters, less significant *p*_FWE_ < 0.001 corrected) and during anticipation of loss (same peak clusters, not significant (*p*_FWE_ = 0.140)).

Within the 22q11DS group, there was no relation between PANSS scores and reward or loss related brain activity.

#### Patients with 22q11DS vs. healthy controls

**Fig. 2:**
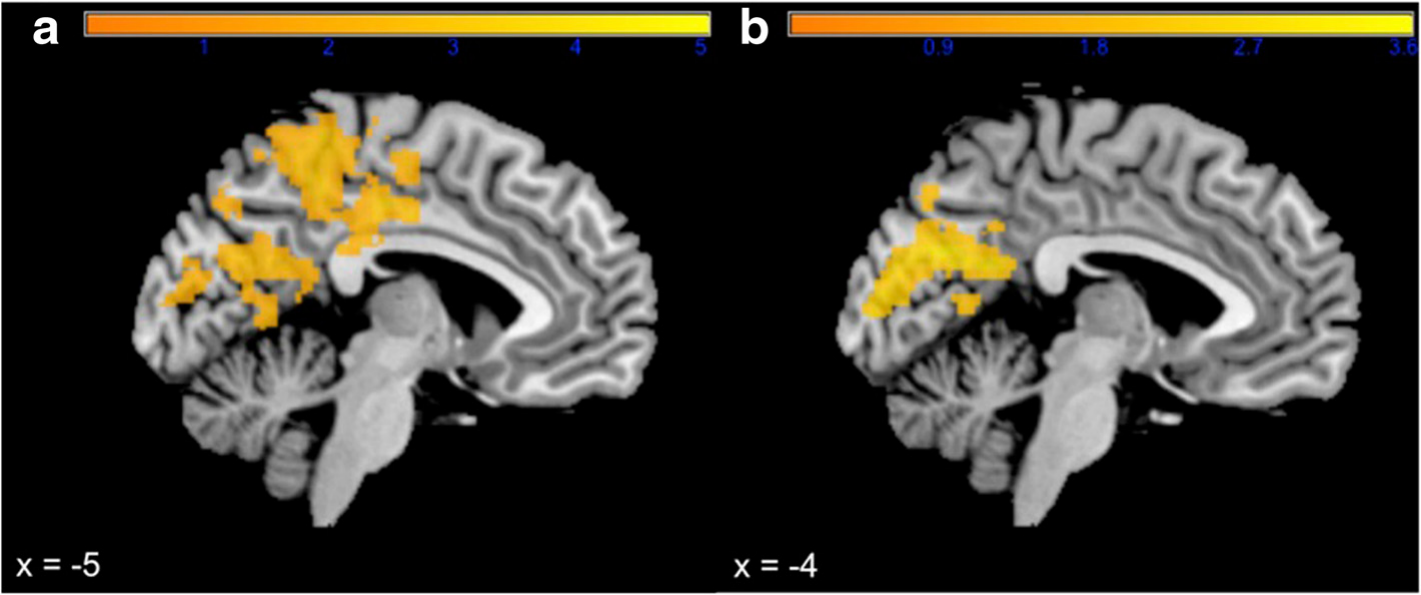
SPM *t* value for healthy controls vs. 22q11DS patients showing significant reduced BOLD activation in 22q11DS patients in the cingulate cortex, primary motor and somatosensory areas during anticipation of reward (**a**) and in posterior cingulate cortex and cuneus during anticipation of loss (**b**)

During *anticipation of reward*, patients with 22q11DS, compared to controls, showed reduced activation (*p*_FWE_ < 0.001) in a cluster (9271 voxels) covering the bilateral cingulate gyrus extending to premotor, primary motor and somatosensory areas (Table 3, Fig. 2a). During *anticipation of loss*, patients with 22q11DS showed reduced activation (*p*_FWE_ < 0.05) in a cluster (3147 voxels) encompassing the left posterior cingulate cortex and extending bilaterally to the cuneus and precuneus (Table 3, Fig. 2b).

**Table 3:** Peak level coordinates in the significant^*^ cluster during anticipation of Loss

Group	Brain structure		BA	MNI coordinates			*T* score
				*x*	*y*	*z*	
22q11DS	L	Cingulate gyrus	24	−6	−6	34	5.29
	L	Cingulate gyrus	24	−10	6	38	4.14
	L	Hippocampus	NA	−28	−22	−8	3.96
	L	Hypothalamus	NA	−8	−6	−10	5.44
	R	Medial frontal gyrus	6	10	0	66	4.14
	L	Middle frontal gyrus	6	−26	−4	64	4.34
	L	Middle frontal gyrus	11	−32	44	−8	4.07
	R	Middle frontal gyrus	6	30	10	60	3.93
	R	Middle frontal gyrus	10	34	38	22	6.04
Controls	R	Cingulate gyrus	24	2	−12	36	6.43
	R	Cingulate gyrus	24	2	−16	44	4.96
	R	Cingulate gyrus	24	10	−12	40	4.12
	L	Insula	13	−32	8	18	4.17
	R	Medial frontal gyrus	6	10	−14	54	4.99
	R	Medial frontal gyrus	6	10	−16	58	4.59
	L	Middle frontal gyrus	11	−30	36	−12	5.62
	R	Middle frontal gyrus	6	26	−18	66	4.75
	R	Middle frontal gyrus	9	28	32	32	4.59
	R	Precentral gyrus	4	20	−26	68	5.29
	R	Precentral gyrus	6	24	−16	74	4.08
	R	Superior temporal gyrus	41	48	−28	8	4.07
22q11DS > controls	No significant results						
Controls > 22q11DS	L	Cuneus	18	−4	−80	24	2.78
	L	Cuneus	18	−4	−90	12	2.74
	L	Cuneus	18	−10	−88	12	2.80
	L	Cuneus	18	−8	−84	20	3.06
	R	Cuneus	18	18	−84	26	3.62
	R	Cuneus	18	10	−82	26	2.84
	R	Cuneus	18	16	−86	16	3.01
	R	Cuneus	7	22	−84	32	2.95
	R	Cuneus	7	22	−80	28	3.00
	L	Middle occipital gyrus	19	−28	−82	14	2.89
	L	Posterior cingulate	23	−4	−54	22	2.86
	L	Precuneus	31	−2	−72	26	3.11
	L	Precuneus	31	−6	−68	24	3.20
	L	Precuneus	31	0	−78	24	2.81
	L	Precuneus	31	−24	−78	14	2.70
	R	Precuneus	7	14	−70	52	2.71

*p < 0.001 FWE-corrected at cluster level

*L* left, *R* right, *BA* Brodmann area

#### 22q11DS Val hemizygotes vs. 22q11DS Met hemizygotes

**Fig. 3:**
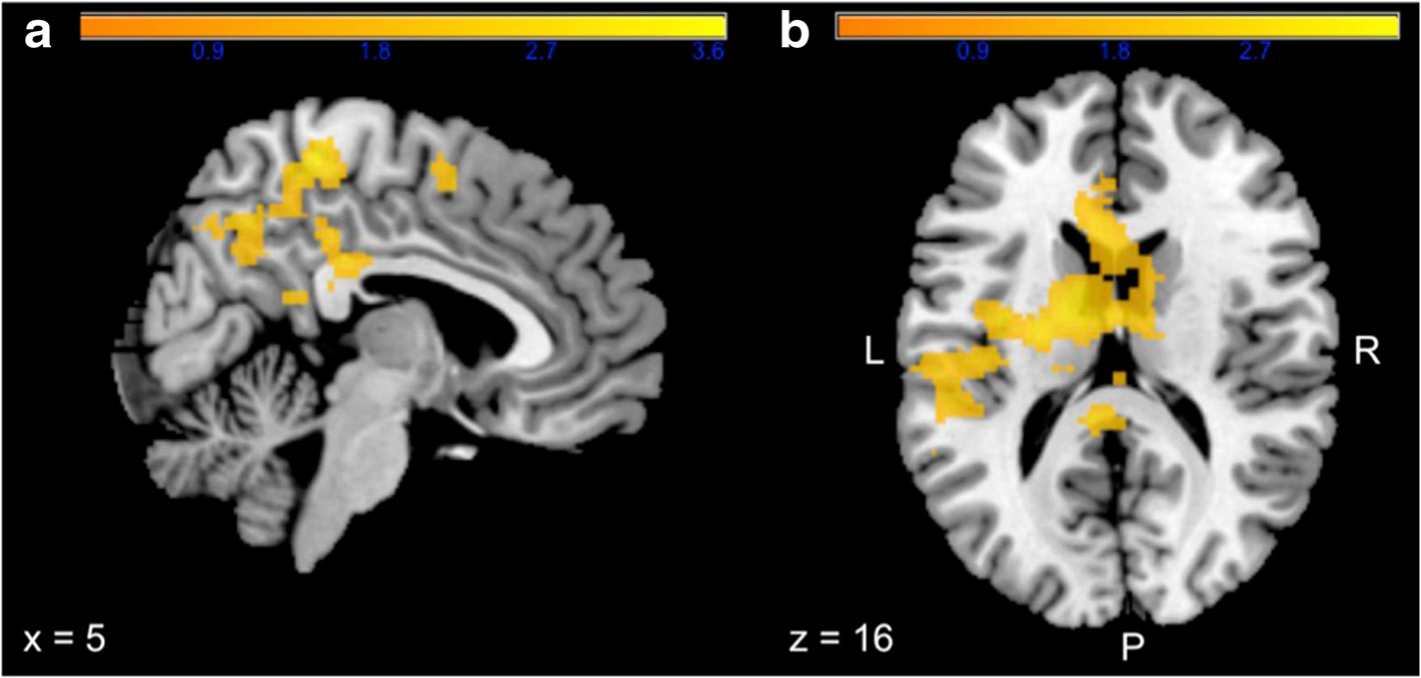
SPM *t* value for 22q11DS Val vs. Met hemizygotes showing significant increased BOLD activation in Val hemizygotes in the cingulate cortex and parietal regions during anticipation of reward (**a**) and reduced activation in anterior cingulate cortex, striatum and insula during anticipation of loss (**b**)

Within the 22q11DS group, anticipation of reward resulted in more activation of the right posterior cingulate and bilateral parietal regions in Val hemizygotes compared to Met hemizygotes (cluster size 3008 voxels, *p*_FWE_ < 0.05, Table 4, Fig. 3a). Anticipation of loss resulted in significantly more activation in the bilateral insula, striatum and left anterior cingulate in Met hemizygotes compared to Val hemizygotes (Cluster size: 4481 voxels, Table 4, Fig. 3b).

**Table 4:** Peak level coordinates in the significant^*^ cluster during anticipation of reward and loss in 22q11DS COMT Val and Met hemizygotes

Reward-neutral							
Group	Brain structure		BA	MNI coordinates			*T* score
				*x*	*y*	*z*	
Val > Met	L	Cingulate gyrus	31	−6	−48	34	2.77
	L	Cingulate gyrus	31	−4	−40	44	2.57
	R	Cingulate gyrus	23	4	−28	28	2.86
	R	Middle frontal gyrus	6	16	−8	62	2.63
	R	Paracentral lobule	4	6	−38	62	3.48
	R	Paracentral lobule	5	8	−42	58	2.99
	L	Postcentral gyrus	3	−20	−32	56	2.93
	R	Postcentral gyrus	3	10	−36	66	3.67
	R	Postcentral gyrus	3	24	−34	58	3.09
	R	Posterior cingulate	30	6	−46	20	2.59
	R	Precentral gyrus	4	32	−32	56	3.24
	R	Precentral gyrus	4	34	−30	68	3.05
	R	Precentral gyrus	4	26	−30	64	3.01
	R	Precentral gyrus	6	22	−24	68	2.73
	L	Precuneus	31	−10	−48	36	2.69
	R	Precuneus	7	12	−66	40	3.00
Met > Val	No significant results						
Loss-neutral							
Group	Brain structure		BA	MNI coordinates			*T* score
				*x*	*y*	*z*	
Val > Met	No significant results			−			
Met > Val	L	Anterior cingulate	24	−2	30	20	2.91
	L	Caudate body	NA	−12	−2	20	3.42
	R	Caudate body	NA	10	−2	20	2.84
	L	Cingulate gyrus	23	−6	−34	28	3.58
	L	Cingulate gyrus	31	−16	−40	28	2.88
	L	Insula	13	−34	−6	16	2.86
	L	Posterior cingulate	23	−2	−40	22	3.29
	L	Putamen	NA	−24	−12	16	2.89
	L	Superior temporal gyrus	22	−64	−42	8	2.79
	L	Thalamus	NA	−18	−8	14	2.73

*p < 0.05 FWE-corrected at cluster level

*L* left, *R* right, *BA* Brodmann area

## Discussion

To our knowledge, this is the first study to investigate the neural substrates of reward processing in people with 22q11DS, a population at high risk of developing a psychotic illness. Our main fMRI findings suggest that reward anticipation in 22q11DS engages a fronto-temporal network. Compared to healthy controls, people with 22q11DS primarily displayed reduced activity in medial frontal regions during reward anticipation. During anticipation of loss, a reduction in bilateral (pre)cuneus and left posterior cingulate activity was observed. Further analyses also revealed an effect of COMT genotype on the 22q11DS reward anticipation network.

### The dysfunctional 22q11DS reward processing network

The 22q11DS reward anticipation network seems different from healthy controls in several ways. During anticipation of reward, reduced activity in the cingulate gyrus and medial frontal brain regions was observed. These are all key structures of the reward circuitry in healthy controls [4, 6, 39, 41–43].

Decreased cingulate gyrus activity during reward anticipation could be related to impairments in predicting reward outcome, since this region is related to prediction error in reinforcement learning [44–46]. Reduced activation in medial frontal brain regions in 22q11DS during reward and posterior cingulate and (pre)cuneus brain regions during loss may be a reflection or consequence of the anatomical abnormalities typically seen in people with 22q11DS. These alterations include grey matter reductions in frontal and temporal regions and widespread white matter reductions primarily in the posterior lobe [47–51].

In contrast to other studies [11, 52], we were not able to find significant activity in the ventral striatum during reward processing, a core region of the reward network [4, 11, 39, 53, 54]. This could be due to the small sample size and the small area that includes the ventral striatum. Moreover, the mixed gender group in our study could have affected the results, since anticipation of monetary reward differentially activates mesolimbic brain regions in women compared to men [55].

Interestingly, similarities in the reward anticipation network exist between 22q11DS and the schizophrenia spectrum. In line with our findings in 22q11DS, previous studies in unmedicated schizophrenia patients showed reduced activity in the cingulate gyrus [49, 56] and a recent study in siblings of schizophrenia patients, at increased genetic risk for schizophrenia, found fronto-striatal dysfunctioning during reward anticipation [57]. Behavioural studies furthermore found evidence for impaired functioning on reward tasks that depend on cortical regions in people with schizophrenia, which is in line with our results and suggested to be associated with negative symptoms [58, 59]. Interestingly, the clinical pattern in 22q11DS is also characterized by predominant negative symptoms [60, 61].

The similarities in the reward network between 22q11DS and schizophrenia spectrum may indicate that 22q11DS is associated with similar behavioural impairments typically seen in schizophrenia such as anhedonia, decreased motivation and a lack of reward sensitivity [11, 62–64]. Future studies should further investigate the presence of these symptoms in relation to the reward processing network in 22q11DS.

Lastly, it is interesting to speculate on the implications of abnormal reward-related activity for the behavioural phenotype in 22q11DS. This may suggest a decreased hedonic component of reward anticipation and, as such, could have implications for (risk of) addiction and substance abuse in 22q11DS [65]. Interestingly, in contrast to schizophrenia patients [66] and the general population, only a small percentage of 22q11DS patients suffer from addiction and display substance abuse [60, 65, 67], possibly suggesting aberrant reward sensitivity. The link between abnormal reward-related brain activity and reward seeking behaviour in 22q11DS requires further investigation.

### COMT genotype effects on 22q11DS reward processing

In line with previous studies investigating reward anticipation with fMRI in healthy controls [19, 68], we found an effect of COMT genotype on reward processing. However, the present results should be considered preliminary due to the small sample size of the COMT genotype subgroups. We observed that the high-activity Val allele compared to Met allele carriers was associated with increased activity in posterior cingulate and parietal regions during anticipation of reward. Whereas the low-activity Met allele, compared to Val allele, was associated with increased activity in the anterior cingulate cortex and striatum during anticipation of loss. These results are in line with previous fMRI research in 22q11DS showing less efficient cingulate activity in Met-carriers, during a response inhibition task [69]. This is furthermore supported by structural findings in 22q11DS adults showing that the COMT Met allele was associated with decreased frontal lobe volume [70], which is consistently found to have abnormal functioning and structure in 22q11DS [47, 49]. While preliminary, these results are noteworthy because they provide clues on the underlying reward-related alterations in neurochemical signaling in 22q11DS, which could lead to more insight in possible treatment targets [71].

Variation in COMT genotype has been associated with altered cortico-striatal dopaminergic activity [72, 73]. 22q11DS COMT hemizygosity has been associated with decreased cortical COMT expression and enzyme activity, possibly greatly increasing extracellular DA in 22q11DS Met-carriers and moderately increasing extracellular DA in 22q11DS Val carriers [24, 25].

Met-hemizygosity in 22q11DS is associated with worse prefrontal cognitive functioning, possibly related to increased levels of tonic DA and decreased phasic DA release [73, 74]. Alterations in DA function have previously also been implicated to play a role in reward-related dysfunction and the development of psychotic symptoms in schizophrenia [3, 7, 75]. Moreover, lower striatal mean D2/3R binding has been found in Met hemizygotes, possibly reflecting higher synaptic DA levels [76]. All in all, these findings may suggest that changes in dopamine function might explain the effect of COMT genotype on reward-related brain activity in frontal and striatal brain regions in 22q11DS. This explanation, however, remains speculative since the mechanism underlying COMT genotype effects on extracellular DA levels is thought to be far more complex because of the different isoforms and the suggested intracellular location of COMT [14, 77–79] and our methods could not provide information on extracellular DA levels.

Lastly, the observation that brain activity associated with anticipation of reward and loss was differentially modulated by COMT genotype in 22q11DS may suggest that COMT genotype impacts preferred reward engagement strategies such as reward and loss seeking or aversion behaviour. This idea is supported by previous work hypothesizing that the Met genotype is associated with higher loss aversion [68] and lower extraversion [80].

### Limitations and future directions

A limitation of the study is the relatively small sample size of the total and COMT specific sample, the presence of psychotic disorder in a part of the 22q11DS group and the use of medication in some subjects, which could have affected brain function [81]. We reanalysed a subset of the 22q11DS group excluding the 22q11DS subjects with psychosis and replicated the majority of our prior fMRI results, finding the same peak clusters in botch conditions. However, in the anticipation of loss condition, the findings did not survive the level of significance, which could be the result of the smaller sample size. The present results should therefore be considered preliminary and replication is needed. In light of the rarity of the disorder and the challenge of recruitment, the sample size of the group however could be considered acceptable.

Future research could address some other limitations of this study. In line with previous studies that used a point scoring system [34], our participants did not receive the actual money that they gained. Lack of a powerful reinforcer such as money might have influenced the participants’ motivation to perform to the best of their abilities, possibly affecting activation patterns in brain reward regions.

Furthermore, given that the BOLD signal is a hemodynamic measure, the neurochemical mechanism behind alterations in the 22q11DS reward network is unclear. The observed between-group and COMT effects could reflect changes in catecholaminergic activity or downstream consequences of these changes on other neurotransmitter systems. Positron emission tomography (PET) studies in this disorder could be an important next step in investigating the degree of dopaminergic abnormalities during reward processing in 22q11DS.

## Conclusions

This study is the first to investigate reward processing in 22q11DS. Our preliminary results suggest that people with 22q11DS engage a fronto-temporal neural network during reward processing and that, compared to controls, brain activation within the 22q11DS group is reduced in medial and frontal brain regions.

Similarities with the reward neural network within the schizophrenia spectrum were observed, which is in line with the clinical overlap between the behavioural impairments typically seen in 22q11DS and schizophrenia.

Our findings may be explained by the anatomical abnormalities typically seen in 22q11DS or by the COMT haplo-insufficiency in 22q11DS, which is hypothesized to result in primarily abnormal frontal DA levels and increased extracellular DA release in low-activity Met hemizygotes. In line with this notion, an effect of 22q11DS COMT genotype on reward processing was additionally observed, which may provide further clues on the underlying reward-related alterations in neurochemical signaling in 22q11DS and its possible relevance for psychotic disorder.

## Abbreviations

22q11DS, 22q11.2 deletion syndrome; BOLD, blood-oxygen-level dependent; COMT, catechol-O-methyl-transferase; DA, Dopamine; fMRI, functional magnetic resonance imaging; GLM, general linear model; IQ, intelligence quotient; MID, monetary incentive delay; PANSS, The Positive and Negative Symptom Scale; WAIS, Wechsler Adult Intelligence Scale

## References

[1] van Os J, Kapur S (2009). Schizophrenia. Lancet.

[2] Howes OD, Kambeitz J, Kim E, Stahl D, Slifstein M, Abi-Dargham A, Kapur S (2012). The nature of dopamine dysfunction in schizophrenia and what this means for treatment. Arch Gen Psychiatry.

[3] Kapur S (2003). Psychosis as a state of aberrant salience: a framework linking biology, phenomenology, and pharmacology in schizophrenia. Am J Psychiatry.

[4] Haber SN, Knutson B (2010). The reward circuit: linking primate anatomy and human imaging. Neuropsychopharmacology.

[5] Berridge KC, Robinson TE (1998). What is the role of dopamine in reward: hedonic impact, reward learning, or incentive salience?. Brain Res Rev.

[6] da Silva AF, Schmitz N, Figee M, Abeling N, Hasler G, van der Meer J, Nederveen A, de Haan L, Linszen D, van Amelsvoort T (2011). Dopaminergic modulation of the human reward system: a placebo-controlled dopamine depletion fMRI study. J Psychopharmacol.

[7] Heinz A, Schlagenhauf F (2010). Dopaminergic dysfunction in schizophrenia: salience attribution revisited. Schizophr Bull.

[8] Jensen J, Willeit M, Zipursky RB, Savina I, Smith AJ, Menon M, Crawley AP, Kapur S (2008). The formation of abnormal associations in schizophrenia: neural and behavioral evidence. Neuropsychopharmacology.

[9] Juckel G, Schlagenhauf F, Koslowski M, Filonov D, Wüstenberg T, Villringer A, Knutson B, Kienast T, Gallinat J, Wrase J, Heinz A (2006). Dysfunction of ventral striatal reward prediction in schizophrenic patients treated with typical, not atypical, neuroleptics. Psychopharmacology (Berl).

[10] Walter H, Kammerer H, Frasch K, Spitzer M, Abler B (2009). Altered reward functions in patients on atypical antipsychotic medication in line with the revised dopamine hypothesis of schizophrenia. Psychopharmacology (Berl).

[11] Juckel G, Schlagenhauf F, Koslowski M, Wüstenberg T, Villringer A, Knutson B, Wrase J, Heinz A (2006). Dysfunction of ventral striatal reward prediction in schizophrenia. NeuroImage.

[12] Juckel G, Friedel E, Koslowski M, Witthaus H, Ozgürdal S, Gudlowski Y, Knutson B, Wrase J, Brüne M, Heinz A, Schlagenhauf F (2012). Ventral striatal activation during reward processing in subjects with ultra-high risk for schizophrenia. Neuropsychobiology.

[13] Roiser JP, Howes OD, Chaddock C, Joyce EM, McGuire P (2013). Neural and behavioral correlates of aberrant salience in individuals at risk for psychosis. Schizophr Bull.

[14] Tunbridge E, Weickert C, Kleinman J, Herman M, Chen J, Kolachana B, Harrison P, Weinberger D (2006). Catechol-o-methyltransferase enzyme activity and protein expression in human prefrontal cortex across the postnatal lifespan. Cereb Cortex.

[15] Chen J, Lipska BK, Halim N, Ma QD, Matsumoto M, Melhem S, Kolachana BS, Hyde TM, Herman MM, Apud J, Egan MF, Kleinman JE, Weinberger DR (2004). Functional analysis of genetic variation in catechol-O-methyltransferase (COMT): effects on mRNA, protein, and enzyme activity in postmortem human brain. Am J Hum Genet.

[16] Malhotra A, Kestler LJ, Mazzanti C, Ph D, Bates JA, Goldberg T, Goldman D (2002). A Functional polymorphism in the COMT gene and performance on a test of prefrontal cognition. Am J Psychiatry.

[17] Gothelf D, Eliez S, Thompson T, Hinard C, Penniman L, Feinstein C, Kwon H, Jin S, Jo B, Antonarakis SE, Morris MA, Reiss AL (2005). COMT genotype predicts longitudinal cognitive decline and psychosis in 22q11.2 deletion syndrome. Nat Neurosci.

[18] Bearden CE, Jawad AF, Lynch DR, Sokol S, Kanes SJ, McDonald-McGinn DM, Saitta SC, Harris SE, Moss E, Wang PP, Zackai E, Emanuel BS, Simon TJ (2004). Effects of a functional COMT polymorphism on prefrontal cognitive function in patients with 22q11.2 deletion syndrome. Am J Psychiatry.

[19] Dreher J-C, Kohn P, Kolachana B, Weinberger DR, Berman KF (2009). Variation in dopamine genes influences responsivity of the human reward system. Proc Natl Acad Sci U S A.

[20] Yacubian J, Kalisch R, Leuenberger B, Sommer T, Schroeder K, Gla J, Braus DF, Bu C (2007). Gene-gene interaction associated with neural reward sensitivity. PNAS.

[21] Bassett AS, Chow EWC, Husted J, Weksberg R, Caluseriu O, Webb GD, Gatzoulis M (2005). Clinical features of 78 adults with 22q11 deletion syndrome. Am J Med Genet.

[22] Murphy KC, Jones L, Owen MJ (1999). High rates of schizophrenia in adults with velo-cardio-facial syndrome. Arch Gen Psychiatry.

[23] Hiroi N, Takahashi T, Hishimoto A, Izumi T, Boku S, Hiramoto T (2013). Copy number variation at 22q11.2: from rare variants to common mechanisms of developmental neuropsychiatric disorders. Mol Psychiatry.

[24] van Beveren NJM, Krab LC, Swagemakers S, Buitendijk G, Buitendijk GHS, Boot E, van der Spek P, Elgersma Y, van Amelsvoort TAMJ (2012). Functional gene-expression analysis shows involvement of schizophrenia-relevant pathways in patients with 22q11 deletion syndrome. PLoS One.

[25] Gothelf D, Law AJ, Frisch A, Chen J, Zarchi O, Michaelovsky E, Ren-Patterson R, Lipska BK, Carmel M, Kolachana B, Weizman A, Weinberger DR (2014). Biological effects of COMT haplotypes and psychosis risk in 22q11.2 deletion syndrome. Biol Psychiatry.

[26] Boot E, Booij J, Zinkstok J, Abeling N, de Haan L, Baas F, Linszen D, van Amelsvoort T (2008). Disrupted dopaminergic neurotransmission in 22q11 deletion syndrome. Neuropsychopharmacology.

[27] Evers LJM, Curfs LMG, Bakker JA, Boot E, da Silva Alves F, Abeling N, Bierau J, Drukker M, van Amelsvoort TAMJ (2014). Serotonergic, noradrenergic and dopaminergic markers are related to cognitive function in adults with 22q11 deletion syndrome. Int J Neuropsychopharmacol.

[28] Matsumoto M, Weickert CS, Beltaifa S, Kolachana B, Chen J, Hyde TM, Herman MM, Weinberger DR, Kleinman JE (2003). Catechol O-methyltransferase (COMT) mRNA expression in the dorsolateral prefrontal cortex of patients with schizophrenia. Neuropsychopharmacology.

[29] Karayiorgou M, Simon TJ, Gogos J (2010). 22q11.2 microdeletions: linking DNA structural variation to brain dysfunction and schizophrenia. Nat Rev Neurosci.

[30] Michaelovsky E, Gothelf D, Korostishevsky M, Frisch A, Burg M, Carmel M, Steinberg T, Inbar D, Apter A, Weizman A (2008). Association between a common haplotype in the COMT gene region and psychiatric disorders in individuals with 22q11.2DS. Int J Neuropsychopharmacol.

[31] Goes FS, McGrath J, Avramopoulos D, Wolyniec P, Pirooznia M, Ruczinski I, Nestadt G, Kenny EE, Vacic V, Peters I, Lencz T, Darvasi A, Mulle JG, Warren ST, Pulver AE (2015). Genome-wide association study of schizophrenia in Ashkenazi Jews. Am J Med Genet Part B Neuropsychiatr Genet.

[32] Ripke S, Neale BM, Corvin A, Walters JTR, Farh K-H, Holmans PA, Lee P, Bulik-Sullivan B, Collier DA, Huang H, Pers TH, Agartz I, Agerbo E, Albus M, Alexander M, Amin F, Bacanu SA, Begemann M, Belliveau RA, Bene J, Bergen SE, Bevilacqua E, Bigdeli TB, Black DW, Bruggeman R, Buccola NG, Buckner RL, Byerley W, Cahn W, Cai G (2014). Biological insights from 108 schizophrenia-associated genetic loci. Nature.

[33] Williams HJ, Owen MJ, O’Donovan MC (2007). Is COMT a susceptibility gene for schizophrenia?. Schizophr Bull.

[34] da Silva AF, Bakker G, Schmitz N, Abeling N, Hasler G, van der Meer J, Nederveen A, de Haan L, Linszen D, van Amelsvoort T (2013). Dopaminergic modulation of the reward system in schizophrenia: a placebo-controlled dopamine depletion fMRI study. Eur Neuropsychopharmacol.

[35] Kay SR, Fiszbein A, Opler LA (1987). The positive and negative syndrome scale (PANSS) for schizophrenia. Schizophr Bull.

[36] Canavan AGM, Dunn G, McMillan TM (1986). Principal components of the WAIS-R. Br J Clin Psychol.

[37] Wechsler D (1997). WAIS-III administration and scoring manual.

[38] Livak KJ (1999). Allelic discrimination using fluorogenic probes and the 5’ nuclease assay. Genet Anal.

[39] Knutson B, Adams CM, Fong GW, Hommer D (2001). Anticipation of increasing monetary reward selectively recruits nucleus accumbens. J Neurosci.

[40] Friston KJ, Frith CD, Turner R, Frackowiak RSJ (1995). Characterizing evoked hemodynamics with fMRI. NeuroImage.

[41] O’Doherty JP, Deichmann R, Critchley HD, Dolan RJ (2002). Neural responses during anticipation of a primary taste reward. Neuron.

[42] Kirsch P, Schienle A, Stark R, Sammer G, Blecker C, Walter B, Ott U, Burkart J, Vaitl D (2003). Anticipation of reward in a nonaversive differential conditioning paradigm and the brain reward system. NeuroImage.

[43] Ernst M, Nelson EE, McClure EB, Monk CS, Munson S, Eshel N, Zarahn E, Leibenluft E, Zametkin A, Towbin K, Blair J, Charney D, Pine DS (2004). Choice selection and reward anticipation: an fMRI study. Neuropsychologia.

[44] Garrison J, Erdeniz B, Done J (2014). Corrigendum to “Prediction error in reinforcement learning: a meta-analysis of neuroimaging studies” [Neurosci. Biobehav. Rev. 37 (7), (2013) 1297–1310]. Neurosci Biobehav Rev.

[45] Rogers RD, Ramnani N, Mackay C, Wilson JL, Jezzard P, Carter CS, Smith SM (2004). Distinct portions of anterior cingulate cortex and medial prefrontal cortex are activated by reward processing in separable phases of decision-making cognition. Biol Psychiatry.

[46] Rushworth MFS, Behrens TEJ (2008). Choice, uncertainty and value in prefrontal and cingulate cortex. Nat Neurosci.

[47] van Amelsvoort T, Daly E, Robertson D, Suckling J, Ng V, Critchley H, Owen MJ, Henry J, Murphy KC, Murphy DG (2001). Structural brain abnormalities associated with deletion at chromosome 22q11: quantitative neuroimaging study of adults with velo-cardio-facial syndrome. Br J Psychiatry.

[48] Kates WR, Burnette CP, Jabs EW, Rutberg J, Murphy AM, Grados M, Geraghty M, Kaufmann WE, Pearlson GD (2001). Regional cortical white matter reductions in velocardiofacial syndrome: a volumetric MRI analysis. Biol Psychiatry.

[49] Chow EWC, Zipursky RB, Mikulis DJ, Bassett AS (2002). Structural brain abnormalities in patients with schizophrenia and 22q11 deletion syndrome. Biol Psychiatry.

[50] Simon TJ, Ding L, Bish JP, McDonald-McGinn DM, Zackai EH, Gee J (2005). Volumetric, connective, and morphologic changes in the brains of children with chromosome 22q11.2 deletion syndrome: an integrative study. NeuroImage.

[51] Antshel KM, Kates WR, Roizen N, Fremont W, Shprintzen RJ (2005). 22Q11.2 deletion syndrome: genetics, neuroanatomy and cognitive/behavioral features keywords. Child Neuropsychol.

[52] Radua J, Schmidt A, Borgwardt S, Heinz A, Schlagenhauf F, McGuire P, Fusar-Poli P (2015). Ventral striatal activation during reward processing in psychosis: a neurofunctional meta-analysis. JAMA Psychiatry.

[53] Grimm O, Vollstädt-klein S, Krebs L, Zink M, Smolka MN (2012). Reduced striatal activation during reward anticipation due to appetite-provoking cues in chronic schizophrenia: a fMRI study. Schizophr Res.

[54] Balodis IM, Potenza MN (2015). Anticipatory reward processing in addicted populations: a focus on the monetary incentive delay task. Biol Psychiatry.

[55] Spreckelmeyer KN, Krach S, Kohls G, Rademacher L, Irmak A, Konrad K, Kircher T, Gründer G (2009). Anticipation of monetary and social reward differently activates mesolimbic brain structures in men and women. Soc Cogn Affect Neurosci.

[56] Nielsen MØ, Rostrup E, Wulff S, Bak N, Lublin H, Kapur S, Glenthøj B (2012). Alterations of the brain reward system in antipsychotic nave schizophrenia patients. Biol Psychiatry.

[57] de Leeuw M, Kahn RS, Vink M (2014). Fronto-striatal dysfunction during reward processing in unaffected siblings of schizophrenia patients. Schizophr Bull.

[58] Gold JM, Waltz JA, Matveeva TM, Kasanova Z, Strauss GP, Herbener ES, Collins AGE, Frank MJ (2012). Negative symptoms and the failure to represent the expected reward value of actions: behavioral and computational modeling evidence. Arch Gen Psychiatry.

[59] Strauss G, Frank M, Waltz J, Kasanova Z, Herbener ES, Gold JM (2011). Deficits in positive reinforcement learning and uncertainty-driven exploration are associated with distinct aspects of negative symptoms in schizophrenia. Biol Psychiatry.

[60] Schneider M, Debbané M, Bassett AS, Chow EWC, Fung WLA, van den Bree MBM, Owen M, Murphy KC, Niarchou M, Kates WR, Antshel KM, Fremont W, McDonald-McGinn DM, Gur RE, Zackai EH, Vorstman J, Duijff SN, Klaassen PWJ, Swillen A, Gothelf D, Green T, Weizman A, Van Amelsvoort T, Evers L, Boot E, Shashi V, Hooper SR, Bearden CE, Jalbrzikowski M, Armando M (2014). Psychiatric disorders from childhood to adulthood in 22q11.2 deletion syndrome: results from the international consortium on brain and behavior in 22q11.2 deletion syndrome. Am J Psychiatry.

[61] Armando M, Girardi P, Vicari S, Menghini D, Digilio MC, Pontillo M, Saba R, Mazzone L, Lin A, Klier CM, Schäfer MR, Amminger GP (2012). Adolescents at ultra-high risk for psychosis with and without 22q11 deletion syndrome: a comparison of prodromal psychotic symptoms and general functioning. Schizophr Res.

[62] Wise RA (1982). Neuroleptics and operant behavior: the anhedonia hypothesis. Behav Brain Sci.

[63] Strauss GP, Waltz J, Gold JM (2014). A review of reward processing and motivational impairment in schizophrenia. Schizophr Bull.

[64] Knutson B, Heinz A (2015). Probing psychiatric symptoms with the monetary incentive delay task. Biol Psychiatry.

[65] Vingerhoets WAM, Van Oudenaren MJF, Van Duin EDA, Bloemen OJN, Booij J, Evers LJM, Boot E, Vergaelen E, Vogels A, Swillen A, Van Amelsvoort TAMJ (2015). P.6.f.005 Prevalence of substance use and the relation with psychosis and catechol-O-methyltransferase in patients with chromosome 22q11 deletion syndrome. Biol Psychiaty Elsevier.

[66] Westermeyer J (2006). Comorbid schizophrenia and substance abuse: a review of epidemiology and course. Am J Addict.

[67] Tang SX, Yi JJ, Calkins ME, Whinna D, Kohler CG, Souders MC, McDonald-McGinn DM, Zackai EH, Emanuel BS, Gur RC, Gur RE (2014). Psychiatric disorders in 22q11.2 deletion syndrome are prevalent but undertreated. Psychol Med.

[68] Schmack K, Schlagenhauf F, Sterzer P, Wrase J, Beck A, Dembler T, Kalus P, Puls I, Sander T, Heinz A, Gallinat J (2008). Catechol-O-methyltransferase val158met genotype influences neural processing of reward anticipation. NeuroImage.

[69] Gothelf D, Hoeft F, Hinard C, Hallmayer JF, Van Dover SJ, Antonarakis SE, Morris MA, Reiss AL (2007). Abnormal cortical activation during response inhibition in 22q11.2 deletion syndrome. Hum Brain Mapp.

[70] van Amelsvoort T, Zinkstok J, Figee M, Daly E, Morris R, Owen MJ, Murphy KC, De Haan L, Linszen DH, Glaser B, Murphy DGM (2008). Effects of a functional COMT polymorphism on brain anatomy and cognitive function in adults with velo-cardio-facial syndrome. Psychol Med.

[71] Boot E, van Amelsvoort TAMJ (2012). Neuroimaging correlates of 22q11.2 deletion syndrome: implications for schizophrenia research. Curr Top Med Chem.

[72] Akil M, Kolachana BS, Rothmond D, Hyde TM, Weinberger DR, Kleinman JE (2003). Catechol-O-methyltransferase genotype and dopamine regulation in the human brain. J Neurosci.

[73] Bilder RM, Volavka J, Lachman HM, Grace AA (2004). The catechol-O-methyltransferase polymorphism: relations to the tonic-phasic dopamine hypothesis and neuropsychiatric phenotypes. Neuropsychopharmacology.

[74] Gothelf D, Schaer M, Eliez S (2008). Genes, brain development and psychiatric phenotypes in velo-cardio-facial syndrome. Dev Disabil Res Rev.

[75] Stopper CM, Floresco SB (2015). Dopaminergic circuitry and risk/reward decision making: implications for schizophrenia. Schizophr Bull.

[76] Boot E, Booij J, Zinkstok JR, Baas F, Swillen A, Owen MJ, Murphy DG, Murphy KC, Linszen DH, Van Amelsvoort T (2011). COMT Val158met genotype and striatal D2/3 receptor binding in adults with 22q11 deletion syndrome. Synapse.

[77] Schott BH, Frischknecht R, Debska-Vielhaber G, John N, Behnisch G, Düzel E, Gundelfinger ED, Seidenbecher CI (2010). Membrane-bound catechol-O-methyl transferase in cortical neurons and glial cells is intracellularly oriented. Front Psychiatry.

[78] Tunbridge EM, Lane TA, Harrison PJ (2007). Expression of multiple catechol-o-methyltransferase (COMT) mRNA variants in human brain. Am J Med Genet Part B Neuropsychiatr Genet.

[79] Tunbridge EM, Harrison PJ, Weinberger DR (2006). Catechol-o-methyltransferase, cognition, and psychosis: Val158Met and beyond. Biol Psychiatry.

[80] Stein MB, Fallin MD, Schork NJ, Gelernter J (2005). COMT polymorphisms and anxiety-related personality traits. Neuropsychopharmacology.

[81] Seeman P (2002). Atypical antipsychotics: mechanism of action. Can J Psychiatry.

