# Erratum to: Neural correlates of reward processing in adults with 22q11 deletion syndrome

**DOI:** 10.1186/s11689-016-9163-8

**Published:** 2016-08-04

**Authors:** Esther D. A. Van Duin, Liesbet Goossens, Dennis Hernaus, Fabiana Da Silva Alves, Nicole Schmitz, Koen Schruers, Therese Van Amelsvoort

**Affiliations:** 1 Department of Psychiatry and Psychology, Maastricht University, Maastricht, The Netherlands; 2 Department of Psychiatry, Academic Medical Centre Amsterdam, Amsterdam, The Netherlands

## Erratum

Upon publication of the original article [1], it was noticed that the legends of Table 2 and Table 3 were incorrect and instead of:

- ‘*p<0.001 FWE-corrected at cluster level
- L left, R right, BA Brodmann area’

**Table 2:** Peak level coordinates in the significant^*^ cluster during anticipation of reward

Group	Brain structure		BA	MNI coordinates			*T* score
				x	y	z	
22q11DS	L	Hypothalamus	NA	−10	−6	−8	4.83
	R	Inferior frontal gyrus	47	26	18	−12	5.08
	L	Medial frontal gyrus	6	−10	−30	74	5.35
	L	Middle frontal gyrus	8	−24	20	48	4.53
	R	Middle frontal gyrus	10	34	50	0	4.53
	L	Middle temporal gyrus	21	−52	−46	4	4.37
	R	Middle temporal gyrus	21	54	−24	−12	4.64
	R	Putamen	NA	28	−10	12	4.64
	L	Superior temporal gyrus	39	−34	−58	28	4.52
	R	Superior temporal gyrus	41	56	−20	4	4.63
Controls	L	Cingulate gyrus	24	−4	−10	40	7.79
	R	Cingulate gyrus	24	4	−12	40	7.26
	R	Cingulate gyrus	23	4	−16	34	6.96
	R	Cingulate gyrus	23	4	−32	28	5.52
	R	Cingulate gyrus	24	2	−18	44	9.02
	R	Cingulate gyrus	23	4	−12	30	5.64
	R	Middle occipital gyrus	18	32	−88	−8	7.39
	L	Posterior cingulate	23	−2	−30	24	9.25
	R	Precentral gyrus	4	20	−28	72	6.22
	L	Precuneus	31	−8	−62	22	5.73
	R	Precuneus	31	20	−78	26	6.58
	R	Superior frontal gyrus	6	6	16	68	5.69
	L	Transverse temporal gyrus	41	−42	−30	12	6.08
22q11DS > controls	No significant results						
Controls > 22q11DS	L	Cingulate gyrus	24	−4	−12	38	3.10
	L	Cingulate gyrus	24	−8	−20	40	3.24
	R	Cingulate gyrus	24	4	−12	40	4.63
	R	Cingulate gyrus	23	4	−30	28	3.28
	R	Cingulate gyrus	24	2	−20	40	5.03
	R	Cingulate gyrus	31	12	−32	42	3.24
	R	Medial frontal gyrus	6	10	−12	74	3.66
	L	Paracentral lobule	5	−8	−44	50	3.12
	R	Paracentral lobule	4	6	−42	72	3.10
	R	Postcentral gyrus	4	12	−38	60	4.60
	L	Precuneus	31	−2	−70	24	3.31

*p<0.05 FWE-corrected at cluster level

L left, R right, BA Brodmann area

**Table 3:** Peak level coordinates in the significant^*^ cluster during anticipation of Loss

Group	Brain structure		BA	MNI coordinates			*T* score
				*x*	*y*	*z*	
22q11DS	L	Cingulate gyrus	24	−6	−6	34	5.29
	L	Cingulate gyrus	24	−10	6	38	4.14
	L	Hippocampus	NA	−28	−22	−8	3.96
	L	Hypothalamus	NA	−8	−6	−10	5.44
	R	Medial frontal gyrus	6	10	0	66	4.14
	L	Middle frontal gyrus	6	−26	−4	64	4.34
	L	Middle frontal gyrus	11	−32	44	−8	4.07
	R	Middle frontal gyrus	6	30	10	60	3.93
	R	Middle frontal gyrus	10	34	38	22	6.04
Controls	R	Cingulate gyrus	24	2	−12	36	6.43
	R	Cingulate gyrus	24	2	−16	44	4.96
	R	Cingulate gyrus	24	10	−12	40	4.12
	L	Insula	13	−32	8	18	4.17
	R	Medial frontal gyrus	6	10	−14	54	4.99
	R	Medial frontal gyrus	6	10	−16	58	4.59
	L	Middle frontal gyrus	11	−30	36	−12	5.62
	R	Middle frontal gyrus	6	26	−18	66	4.75
	R	Middle frontal gyrus	9	28	32	32	4.59
	R	Precentral gyrus	4	20	−26	68	5.29
	R	Precentral gyrus	6	24	−16	74	4.08
	R	Superior temporal gyrus	41	48	−28	8	4.07
22q11DS > controls	No significant results						
Controls > 22q11DS	L	Cuneus	18	−4	−80	24	2.78
	L	Cuneus	18	−4	−90	12	2.74
	L	Cuneus	18	−10	−88	12	2.80
	L	Cuneus	18	−8	−84	20	3.06
	R	Cuneus	18	18	−84	26	3.62
	R	Cuneus	18	10	−82	26	2.84
	R	Cuneus	18	16	−86	16	3.01
	R	Cuneus	7	22	−84	32	2.95
	R	Cuneus	7	22	−80	28	3.00
	L	Middle occipital gyrus	19	−28	−82	14	2.89
	L	Posterior cingulate	23	−4	−54	22	2.86
	L	Precuneus	31	−2	−72	26	3.11
	L	Precuneus	31	−6	−68	24	3.20
	L	Precuneus	31	0	−78	24	2.81
	L	Precuneus	31	−24	−78	14	2.70
	R	Precuneus	7	14	−70	52	2.71

*p<0.05 FWE-corrected at cluster level

L left, R right, BA Brodmann area

should both read:

- ‘*p<0.05 FWE-corrected at cluster level
- L left, R right, BA Brodmann area’

This has now been corrected in this erratum and is shown in the below tables.

## References

[1] van Duin EDA, Goossens L, Hernaus D, da Silva Alves F, Schmitz N, Schruers K, van Amelsvoort T (2016). Neural correlates of reward processing in adults with 22q11 deletion syndrome. J Neurodev Disord.

